# Aberrant FGFR4 signaling worsens nonalcoholic steatohepatitis in FGF21KO mice

**DOI:** 10.7150/ijbs.58776

**Published:** 2021-06-22

**Authors:** Youxi Yu, Xiaoju Shi, Qianqian Zheng, Xingtong Wang, Xingkai Liu, Min Tan, Guoyue Lv, Ping Zhang, Robert C. Martin, Yan Li

**Affiliations:** 1 Department of Surgery, School of Medicine, University of Louisville, Louisville, KY 40202, USA.; 2 Department of Hepatobiliary and Pancreatic Surgery, The First Hospital of Jilin University, Changchun 130021, China.; 3 Department of Pathophysiology, Basic Medicine College, China Medical University, Shenyang 110122, China.; 4 Department of Tumor Center, The First Hospital of Jilin University, Changchun, Jilin, China.

**Keywords:** Fibroblast growth factor 15/19, Fibroblast growth factor 21, Nonalcoholic steatohepatities, Nonalcoholic fatty liver disease, Bile acid.

## Abstract

**Background:** Nonalcoholic steatohepatitis (NASH) is the most severe form of non-alcoholic fatty liver disease (NAFLD) and a potential precursor of hepatocellular carcinoma (HCC). In our previous studies, we found that endocrine fibroblast growth factor 21 (FGF21) played a key role in preventing the development of NASH, however, the FGF15/19 mediated-FGFR4 signaling worsened NASH and even contributed to the NASH-HCC transition. The aim of this study is to determine whether FGF15/FGFR4 signaling could alleviate or aggravate NASH in the FGF21KO mice.

**Methods:** NASH models were established in FGF21KO mice fed with high fat methionine-choline deficient (HFMCD) diet to investigate FGF15/FGFR4 signaling during early stage NASH and advanced stage NASH. Human hepatocytes, HepG2 and Hep3B cells, were cultured with human enterocytes Caco-2 cells to mimic gut-liver circulation to investigate the potential mechanism of NASH development.

**Results:** Significant increase of FGF15 production was found in the liver of the NASH-FGF21KO mice, however the increased FGF15 protein was unable to alleviate hepatic lipid accumulation. In contrast, up-regulated FGF15/19/FGFR4 signaling was found in the FGF21KO mice with increased NASH severity, as evident by hepatocyte injury/repair, fibrosis and potential malignant events. In *in vitro* studies, blockage of FGFR4 by BLU9931 treatment attenuated the lipid accumulation, up-regulated cyclin D1, and epithelial-mesenchymal transition (EMT) in the hepatocytes.

**Conclusion:** The increased FGF15 in NASH-FGF21KO mice could not substitute for FGF21 to compensate its lipid metabolic benefits thereby to prevent NASH development. Up-regulated FGFR4 signaling in NASH-FGF21KO mice coupled to proliferation and EMT events which were widely accepted to be associated with carcinogenic transformation.

## Introduction

Nonalcoholic steatohepatitis (NASH) is the most severe form of non-alcoholic fatty liver disease (NAFLD) and a potential precursor of hepatocellular carcinoma (HCC) 1. NAFLD advances to the progressive form of NASH in about 44% of patients 2. HCC has been recognized increasingly in NASH patients before the cirrhosis stage 3, 4. The basis of NASH-related HCC carcinogenesis remains largely unknown; however, early treatment may determine long-term NASH prognosis 5. Our previous studies showed that up-regulation of fibroblast growth factor (FGF)15/19 signaling and its receptors FGFR4/beta-klotho in NASH-HCC mice and in HCC patients 6, 7. We also found that lack of FGF21 increased NASH severity and ensued pro-inflammatory signaling in the FGF21 knockout (FGF21KO) mice 8. In a diabetes-HCC mouse model, we found that hepatic FGF21 protein level increased in steatohepatitis but decreased during the development of HCC 9. Our previous studies indicated that FGF21 played a key role in preventing the development of the major characteristics of NASH: steatosis, inflammation, and metabolic syndrome, however, the FGF15/19 mediated-FGFR4 signaling worsened NASH and even contributed to the NASH-HCC transition.

Fibroblast growth factors are a group of structurally related polypeptides, involved in various biological processes such as neuronal functions, development, differentiation, and metabolism 10. There are three endocrine FGFs--FGF15/19, FGF21, and FGF23--identified in mouse/human, and human FGF19 is the orthologous gene of mouse FGF15 11. FGF21 is predominantly produced by hepatocytes, while FGF15/19 is mostly secreted from the ileum but targeted to liver. Under the regulation of peroxisome proliferator-activated receptor α (PPARα) in response to the accumulation of lipids, hepatic FGF21 elicits metabolic benefits, in turn acting on the distal adipose tissue adipocytes, through the transmembrane receptor FGFR1-coreceptor β-Klotho complex 12. This major endocrine action of FGF21 results in a combination of effects including control of lipolysis, clearance of excessive FFAs, enhancing expenditure of the stored lipid energy by mitochondrial substrate oxidation, catabolism and uncoupling, and therefore, negatively regulating hepatic or tissue steatosis, and adiposity 13-15. As FGF21 acts as endocrine hormones and take part in the regulation of glucose and lipid metabolism 16, pharmacological application of FGF21 holds great promise as effective therapeutic means for treating NASH), obesity and diabetes^17^-^19^. FGF15/FGF19 has been also reported to prevent NASH 20-22. Although FGF15/19 upon the mitogenic and cytoprotective effects is critical in protection of hepatocyte from lipid-mediated cellular stress and injury 20, the carcinogenetic role of FGF15/19/FGFR4 signaling has been recognized in various cancers, including breast, gastric, lung, prostate, nasopharyngeal carcinoma and liver cancer 23. FGF15/19 on glucose metabolism and its crucial regulatory role in bile acid (BA) homeostasis have endorsed FGF15/19's metabolic benefits for whole body lipid metabolism 24. However, it is not known whether FGF19 signaling in the liver is indispensable against hepatic lipid accumulation to substitute for FGF21 to compensate the metabolic benefits when FGF21 protein is compromised in liver.

In this study, early and advanced NASH models were established in FGF21KO mice fed with high fat methionine-choline deficient (HFMCD) diet to investigate FGF15/FGFR4 signaling during NASH development. We sought to determine whether FGF15/FGFR4 signaling could alleviate or aggravate NASH in FGF21KO mice challenged with HFMCD.

## Materials and methods

### Establishing NASH models

Male FGF21 Knockout (FGF21KO) mice with C57 BL/6J background were generously granted by Dr. Steve Kliewer (University of Texas Southwestern Medical Center). Wild-type (WT) C57 BL/6J mice obtained from Jackson Laboratory (Bar Harbor, ME). Six-weeks old male mice were fed with Rodent Diets, HFMCD (L-amino acid diet with 60 kcal% fat, 0.1% methionine and no added choline, A06071302, Research Diets, Inc., New Brunswick, NJ) to induce NASH. Rodent Diet (CD, 10% kcal% fat, D12450B, Research Diets, Inc., New Brunswick, NJ) was used as control diet (CD). Both FGF21KO and WT mice with respective diets were assigned randomly into the groups: WT-CD; WT-HFMCD; FGF21KO-CD; FGF21KO-HFMCD. The mice were sacrificed at week 2 for early NASH model and 3 months for advanced NASH model according to previous report 25. Body weight, liver weight and gross anatomy of liver lobes were determined and evaluated. Serum and hepatic tissues were harvested for further biochemical analysis. Serum alanine aminotransferase (ALT) was measured using an ALT infinity enzymatic assay kit (ThermoFisher Scientific Inc., Waltham, MA). Serum and hepatic triglyceride (TG) was determined using a mouse TG assay kit (Cayman Chemical Company, CA). The animal procedures were approved by the Institutional Animal Care and Use Committee of University of Louisville, which is certified by the American Association for Accreditation of Laboratory Animal Care.

### Gross anatomy, histopathological examination and NASH scoring

The whole liver was isolated, weighted, and examined macroscopically for each animal. The harvested tissues were fixed in 10% neutral phosphate buffered formalin or embedded in Optimal Cutting Temperature medium (OCT) for liquid nitrogen frozen. The formalin fixed tissues were further embedded in paraffin and sectioned to a thickness of 5 µm for histological and immunohistochemical examinations. Oil Red O staining for lipid accumulation in the liver tissues was performed in OCT-embedded frozen tissue. Hematoxylin-and-eosin (H&E) staining for histology was performed in paraffin-embedded frozen tissue. The images were reviewed and analyzed microscopically for determination of NASH. The Histological Scoring System for NASH is reported by the Pathology Subcommittee of the NASH Clinical Research Network 26. This scoring system is calculated by the sum of scores of steatosis (0-3), lobular inflammation (0-3) and hepatocyte ballooning (0-2). The scoring is conducted as follows: Steatosis: 0, <5%; 1, 5-33%; 2, >33%; 3, > 66. Lobular Inflammation: 0, no foci; 1, <2 foci/200X; 2, 2-4 foci/200X; 3, >4 foci/200X. Hepatocyte Ballooning: 0, no balloon cells; 1, 1-5 balloon cells/200X; 2, >5 balloon cells/200X.

### Immunohistochemical (IHC) analysis

IHC staining was carried out on the paraffin-embedded tissues using the DAKO EnVisionTM+System Kit (DAKO Corporation, Carpinteria, CA) according to the manufacturer's instructions. Double-IHC staining was performed on the paraffin embedded tissue sections. In brief, endogenous peroxidase was blocked with 3% hydrogen peroxide, and then with 5% BSA for 30 min to block non-specific reaction. These tissue sections were incubated with the first-primary antibodies (see antibody list in supplemental) over night. Tissue sections were incubated with AP-conjugated polymer (1: 300-400 dilutions with PBS) for 1 hour in room temperature, and then incubated with mixture of AP-substrate, AP-activator and AP-chromogen (Abcam, Cambridge, MA) to develop pink color. Tissue sections were then incubated with the second-primary antibodies (see antibody list in supplemental Table 1) for 2 hours in room temperature. Tissue sections were incubated with HRP-conjugated polymer (1: 300-400 dilutions with PBS) for 1 hour in room temperature. Hematoxylin staining was performed before emerald-chromogen staining (Abcam, Cambridge, MA). Tissue sections and then incubated with emerald-chromogen to develop green color. Digital images were acquired with the Olympus 1×51 microscope (Olympus, Pittsburgh, PA) at 10x magnification using the Olympus DP72 digital camera and the length of scratch-wound was measured via the cellSens Dimention imaging system. Computer image analysis was performed, and the acquired color images from the immunohistochemical staining were defined a standard threshold according to the software specification. The computer program then quantified the threshold area represented by color images.

### Terminal deoxynucleotidyl transferase-mediated dUTP nick end labeling (TUNEL) assay

A TUNEL assay was performed using an Apop-Tag Peroxidase *In Situ* Apoptosis Detection Kit (Chemicon, Billerica, CA). Briefly, the deparaffinized and rehydrated tissue sections were treated with proteinase K (20 mg/L) for 15 min, and then incubated with terminal deoxynucleotidyl transferase (TdT) and digoxigenin-11-dUTP for 1 hr at 37°C. Anti-digoxigenin antibody conjugated with horseradish peroxidase (HRP) along with the substrate (DAB-H_2_O_2_) was used for visualization. Apoptosis was quantitatively analyzed by counting the TUNEL positive cells in ten fields for each section at 20X magnification. The apoptotic index was presented as TUNEL positive cells per 100 cells.

### Western blot assay

The protein levels for the biomarkers were semi-quantified by Western blot analysis as described previously 25. Electrophoresis was performed on 12% SDS-PAGE gel and the separated proteins were transferred to nitrocellulose membrane. The membranes were incubated with the primary antibodies (see supplemental Table 1 antibody list) overnight at 4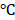 and with secondary antibody for 1 hour at room temperature. The antigen-antibody complexes were then visualized using ECL kit (Amersham, Piscataway, NJ). The protein bands were quantified by densitometry analysis.

### Real-Time RT-PCR (qPCR)

Total RNA was extracted using the TRIzol reagent (Invitrogen). First-strand complimentary DNA (cDNA) was synthesized from total RNA, according to manufacturer's protocol from the RNA PCR kit (Promega, Madison, WI, USA). Quantitative PCR was carried out using the ABI 7300 real-time PCR system (Applied Biosystems, Carlsbad, CA). The primers are listed in supplemental Table 2. The target mRNA expression was quantified and β-actin was used as an endogenous reference. Results were expressed as fold change in gene expression.

### Cell lines and *in vitro* study

The cells for *in vitro* study include a mouse hepatic cell line, FL83B (ATCC^®^ CRL-2390), human HCC cell lines, HepG2 (ATCC^®^ HB-8065) and Hep3B (ATCC^®^ HB-8064), and a human colorectal adenocarcinoma cell line, Caco-2 (ATCC^®^ HTB-37). The cells were cultured, FL83B cells in the F12K medium (ATCC), HepG2 and Hep3B cells in DMEM medium, and Caco-2 cells in EMEM medium respectively, with 10-15% fetal bovine serum. To study the effects of FFA on the cell lines regarding the FGF15/FGFR4 signaling, palmitate (PA) media (Sigma, P9767), recombinant mouse (rm) FGF-15 protein (Abcam, ab125734), recombinant human (rh) FGF21 protein (Abcam, ab217404) and BLU9931 (MedChemExpress, HY-12823), and rhFGF19 (R&D, 969-FG) were used to treat the cells. PA media was made by dissolving 2% bovine serum albumin (BSA, US Biologicals, A1311) in cell culture medium and the 100uM PA working solution was prepared from a high concentration (20 mM) stock PA solution made by dH2O heated to 70° C. Based on the previous reports, rmFGF15 protein was applied at 100ng/ml 27, rhFGF21 protein was applied at 1.1µg/ml 28, BLU9931 was applied at the concentration of 100nM 29, for up to 24 hours. rhFGF19 was applied at the concentration of 100ng/ml 27, for up to 24 hours. Caco-2 cells were co-cultured with HepG2 or Hep3B cells for up to 24 hours to study the FGFR4 signaling based cross-talk between enterocyte and hepatocytes. Immunofluorescent staining was performed in the cells using FITC-conjugated or PE-conjugated IgG (see supplemental Table 1 antibodies list) and DAPI for counterstaining.

### Statistical analysis

Collected data from repeated experiments were presented as mean ± SD. Statistical analysis was performed by using SPSS V.17.0. Statistical significance was determined by ANOVA. The post hoc Tukey's test was used for analysis of any differences between groups. Group difference was considered significant for p < 0.05 (*), p < 0.01(**).

## Result

### Lack of FGF21 worsens the HFMCD-induced NASH in mice

**Figure 1:**
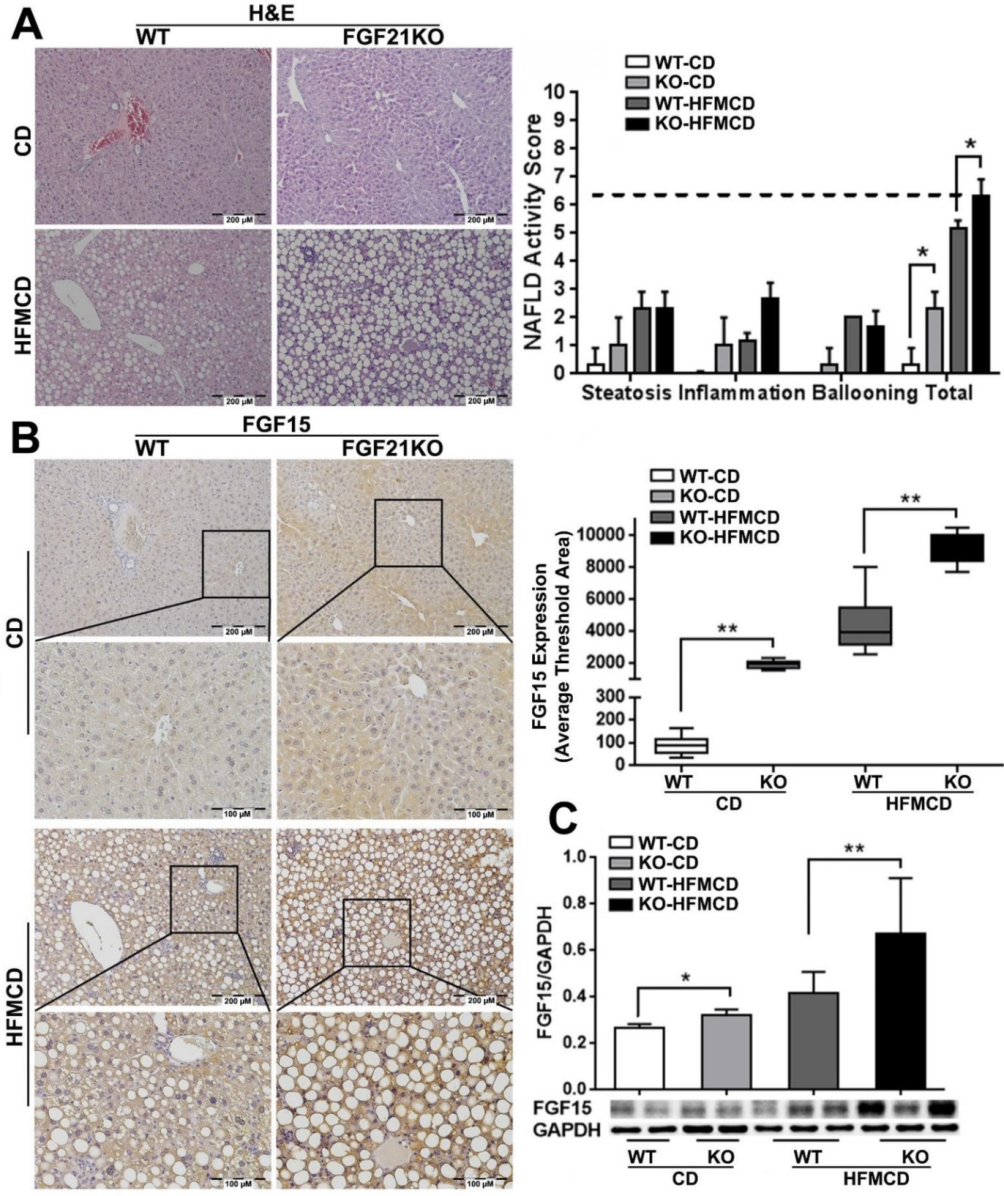
** A:** Representative histology by H&E staining and NAFLD activity score (NAS) in the liver tissues from early NASH model and controls. For histological details, bland steatosis was characterized as wildly distributed lipid drops being detected while steatohepatitis was characterized as infiltration of inflammatory cells in the acinar zone and in the form of hepatocyte ballooning being detected. **B:** Representative images of FGF15 expression by IHC staining along with computer-imaging analysis. C: FGF15 expression by Western blot analysis in the liver tissues from early NASH model and controls. WT: wild type; KO: FGF21KO; CD: control diet; and HFMCD: high fat methionine-choline deficient. *, p<0.05; **, p<0.01.

Based on the previous reports 25, we established an early NSAH model in FGF21KO mice with 2-weeks HFMCD feeding. The gross appearance of NASH liver, unlike the normal liver lobes with red-velvet color, showed diffusely pale-yellow-tan color lobes (Figure S1A) along with increases of liver weight, body weights, glucose tolerance, and serum ALT level in FGF21KO-HFMCD mice (Figure S1B). Increased FGF21 expression by IHC was found in HFMCD feeding WT mice but undetected in FGF21KO mice (Figure S1C). Steatohepatitis was defined in mice, as evident by the histology and confirmed by NAFLD Active Score (NAS) system which was accepted as a surrogate for the histologic diagnosis of NASH. As previously reported in patients with NAFLD, the score of ≥ 5 strongly correlated with a diagnosis of “definite NASH” whereas the score ≤ 3 correlated with a diagnosis of “not NASH 26. All the mice with 2-weeks HFMCD feeding were found with NAS of > 5 and diagnosed as steatohepatitis. The highest NAS was found in the group of FGF21KO-HFMCD mice, with statistical significance compared to all other groups (Figure 1A). Consistent to NAS, highest protein level of FGF15 by IHC was found in the group of FGF21KO-HFMCD mice, with statistical significance compared to all other groups (Figure 1B). Western blot analysis confirmed the IHC results (Figure 1C). The results indicated that FGF15 protein was significantly increased the liver tissues of FGF21KO mice, however the increased FGF15 protein did not show protection against the HFMCD induced steatohepatitis in FGF21KO mice.

### Ileum FGF15 upregulates hepatic FGFR4-β‑klotho in FGF21KO-HFMCD mice

**Figure 2:**
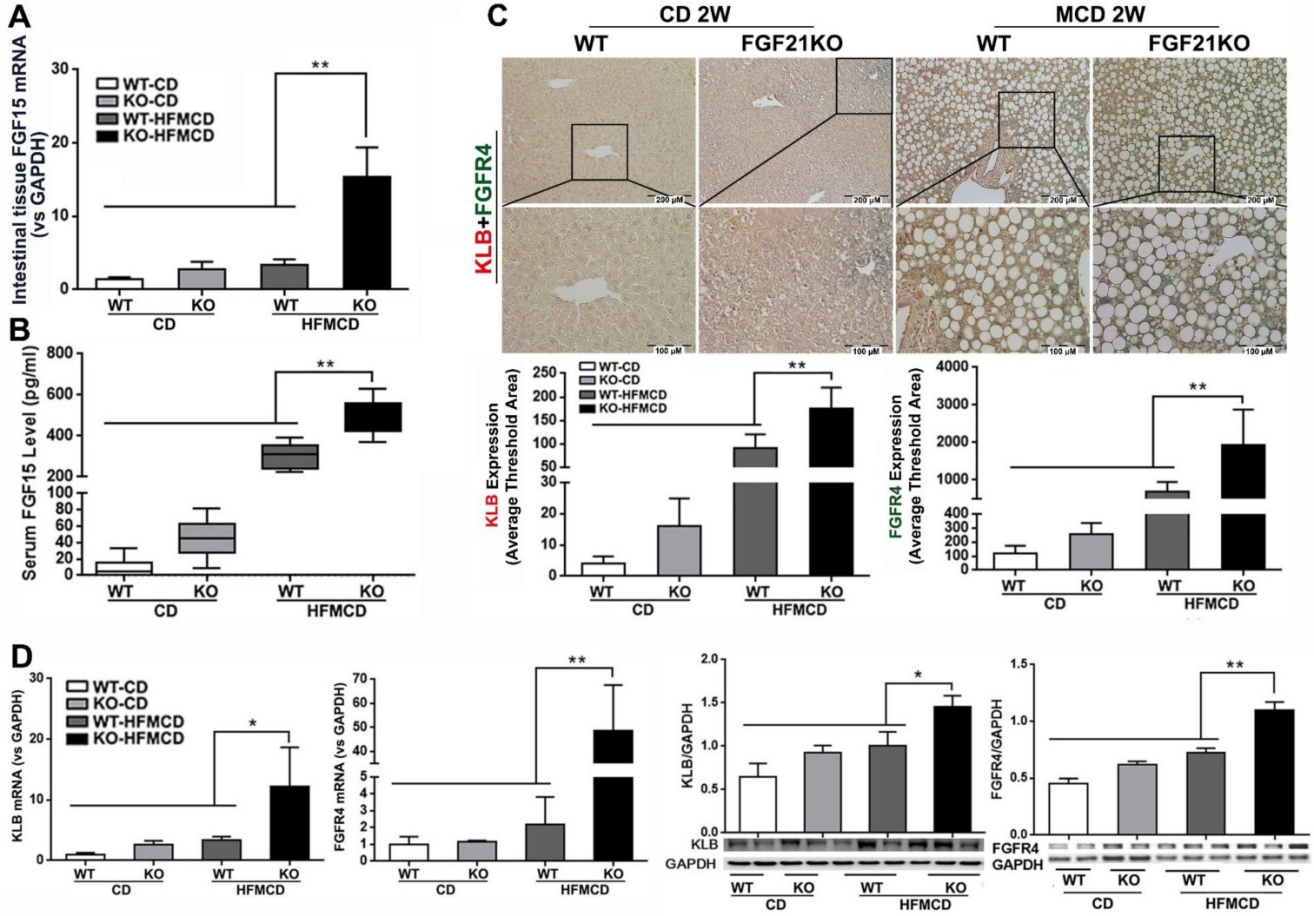
**A-B:** FGF15 mRNA expression by qPCR in the ileum tissues and FGF15 protein levels by ELISA assay in serum from early NASH model and controls. **C:** Representative images of FGFR4/β-klotho expression by a dual IHC staining along with computer-imaging analysis in liver tissues from early NASH model and controls. **D:** FGFR4/β-klotho mRNA expression by qPCR and FGFR4/β-klotho protein levels by Western blot in the liver tissues from early NASH model and controls. KLB: β-klotho; WT: wild type; KO: FGF21KO; CD: control diet; and HFMCD: high fat methionine-choline deficient. *, p<0.05; **, p<0.01.

FGF15/19 is an enterokine and expresses abundantly in the distal small intestine. Upon bile acids stimulation, FGF15/19 reaches liver via the portal blood and binds to FGFR4 and co-receptor β-klotho, triggering a signaling cascade involving hepatic bile acid, lipid and glucose metabolism 30, 31. FGF15/19 is not physiologically expressed in the liver 32, but pathological FGF19 expression in liver tissues was detected in patients with hepatitis C virus-related cirrhosis or biliary cirrhosis 33. We further investigated the resource of FGF15 production and hepatic FGFR4/β-klotho expressions. The results indicated that significantly increased mRNA expression in the intestinal tissues and increased serum FGF15 protein levels were found in FGF21KO-HFMCD mice, compared to all other groups (Figure 2A-B). However, the mRNA of FGF15 was not detectable in hepatic tissues of all groups of mice (data not shown). A dual IHC staining for FGFR4 and β-klotho was performed in the liver tissues. The results indicated that FGFR4 and β-klotho were co-expressed in the hepatocytes, while FGFR4/β-klotho expressions were significantly up-regulated in the FGF21KO-HFMCD mice, compared to all other groups (Figure 2C). qPCR and Western blot analysis of liver tissues further confirmed the IHC results (Figure 2D). The results indicated that the hepatic FGFR4/β-klotho signaling was significantly upregulated in the FGF21KO-HFMCD mice, implying that severity of NASH might associate to the aberrant FGFR4/β-klotho signaling.

### FGF15 is unable to alleviate steatosis but up-regulates FGFR4 in the FGF21KD hepatocytes

**Figure 3:**
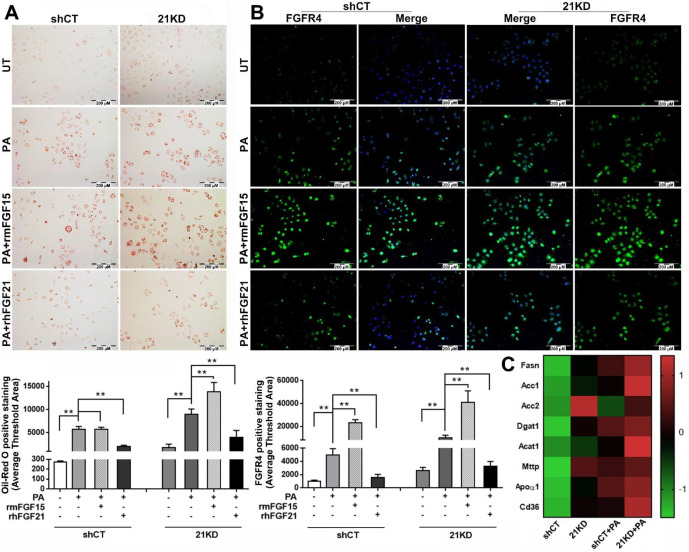
** A:** Lipid accumulation detected by Oil Red O staining along with computer-imaging analysis in shCT-FL83B cells and FGF21KD-FL83B cells challenged with PA (palmic acid) at 100uM for 48 hours, and treated with rmFGF15 at 100ng/ml and rhFGF21 at 1.1µg/ml. **B:** FGFR4 expression detected by immunofluorescent staining in shCT-FL83B cells and FGF21KD-FL83B cells challenged with PA at 100uM for 24 hours, and treated with rmFGF15 at 100ng/ml and rhFGF21 at 1.1µg/ml. **C:** Alterations of lipid metabolic enzymes in shCT-FL83B cells and FGF21KD-FL83B cells challenged with PA at 100uM for 24 hours. FASN: Fatty Acid Synthase; Acc1: acetyl-coenzyme A carboxylase 1; Acc2: acetyl-coenzyme A carboxylase 2; Dgat1: Diacylglycerol acyltransferases 1; Acat1: Acyl-CoA:cholesterol acyltransferase 1; Mttp: Microsomal triglyceride transfer protein; Apoα1: apolipoprotein α-1. UT: untreated; PA: palmic acid; shCT: shCT-FL83B cells; 21KD: FGF21KD-FL83B cells. **, P<0.01.

The binding of FGF15/19 to FGFR4/β-Klotho not only suppresses BA synthesis in hepatocytes via inhibition of cholesterol 7α-hydroxylase 1 (CYP7A1), the rate-limiting step for bile acid synthesis^34^, but also activates signaling cascades leading to increased insulin sensitivity, improved glucose metabolism, and body weight reduction while on a high-fat diet^35^. However, it is unknown whether FGF15 can directly alleviate steatosis in hepatocytes. Therefore, we further investigated the effect of FGF15 on steatosis using FL83B cells, a benign mouse cell line of hepatocyte. To mimic the FGF21KO mice, a shRNA assay was performed to knockdown (KD) FGF21 gene in the FL83B cells. Both FGF21KD (21KD) FL83B cells and the shRNA control (shCT) FL83B cells were challenged with palmitic acid (PA) and treated with rmFGF15 or rhFGF21. Lipid accumulation in hepatocytes were detected by Oil-red O staining. The result indicated that PA challenging significantly up-regulated lipid accumulation in both FGF21KD FL83B cells and shCT-FL83B cells, while treatment with rhFGF21 attenuated the up-regulated lipid accumulation. Unlike FGF21, FGF15 treatment did not show the attenuation of up-regulated lipid accumulation in the shRNA control FL83B cells. In contrast, highest level of lipid accumulation was found in the FGF21KD-FL83B cells with FGF15 treatment (Figure 3A). As a regulatory function of hepatic FGFR4 to promote hepatic TG accumulation has been reported previously 36, we further investigated the FGFR4 levels in hepatocytes. FGF15 treatment significantly up-regulated FGFR4 expression in both shCT-FL83B and FGF21KD-FL83B cells challenged with PA, however, FGF21 treatment did not show up-regulation of FGFR4 levels (Figure 3B). We further investigated the major enzymes for *de novo* synthesis, Fatty acid (FA) esterification and FA transport. Up-regulated FASN, Acc1 and Acc2 (*de novo* synthesis), Dgat1 and Acat1 (esterification), and Mttp, Apoα1 and CD36 (transport) were found in FGF21KD-FL83B cells challenged with PA (Figure 3C), implying that the increased TG storage in hepatocytes could be either from *de novo* synthesis or FFAs uptake. Taken together, the bioactivities of FGF15 on hepatocytes, instead of alleviating lipid accumulation, was shown to up-regulate FGFR4, while compromised FGF21 worsened steatosis in hepatocytes.

### NASH progression is associated with the up-regulated FGFR4 levels in FGF21KO mice

**Figure 4:**
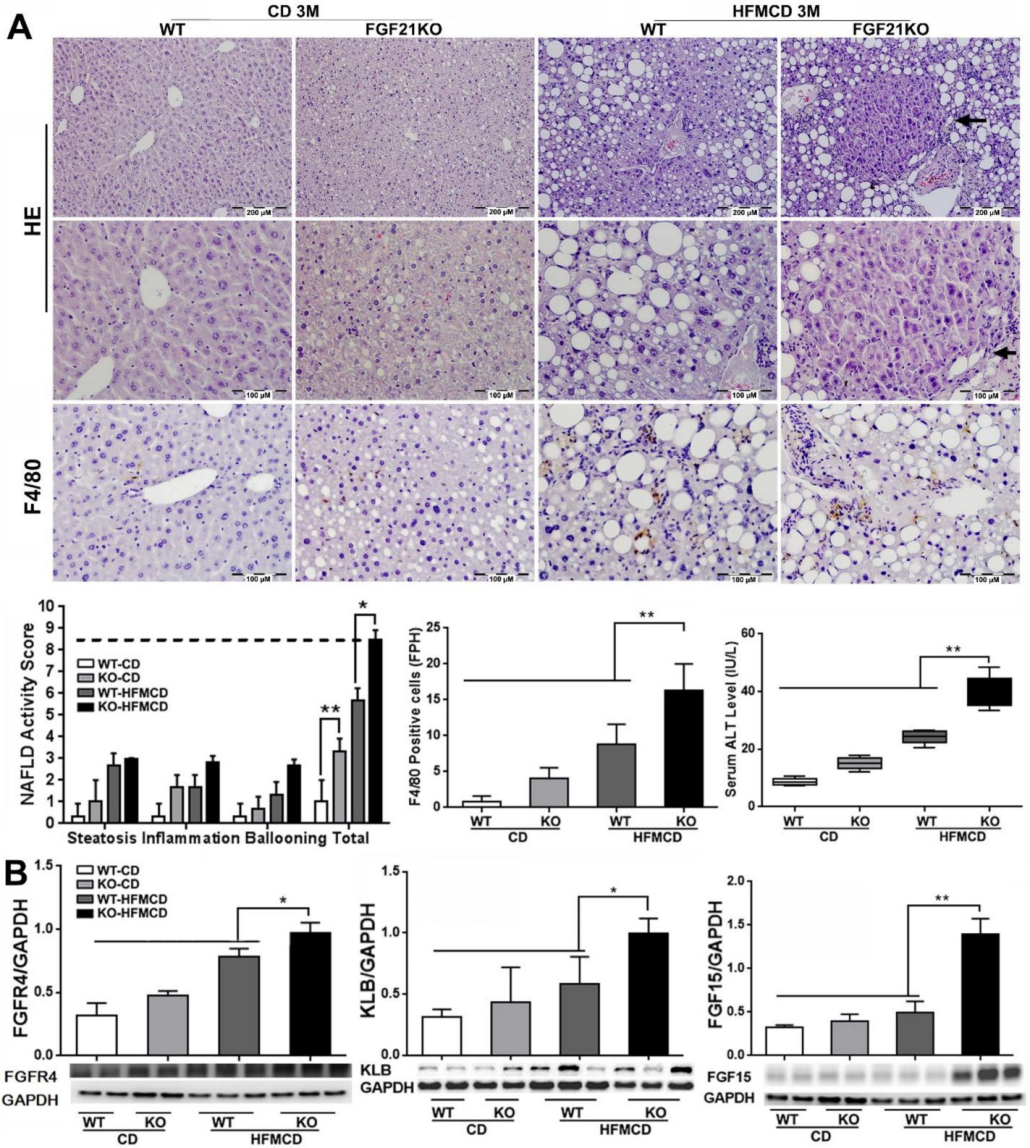
** A:** Representative histology by H&E staining and Kupffer cells/macrophages detected by F4/80 staining in the liver tissues from advanced NASH model and controls. NAFLD activity score (NAS) and index of F4/80 positive cells were calculated based on H&E and F4/80 staining. Arrow: a nodule was detected in the section by H&E stain in hepatic parenchyma from FGF21KO+HFMCD mice. **B:** The protein levels of FGFR4, β-klotho and FGF15 by Western blot in the liver tissues from advanced NASH model and controls. KLB: β-klotho; WT: wild type; KO: FGF21KO; CD: control diet; and HFMCD: high fat methionine-choline deficient. FPH: field per high power. *, p<0.05; **, p<0.01.

Although the FGFR4 mediated-benign hepatic TG storage might provide protection on hepatocytes, continuously up-regulated FGFR4 signaling could play a deleterious role contributing to cell proliferation and progression of cancers 23. According to this hypothesis, we further determined the levels of FGF15 as well its receptors FGFR4/β-klotho (KLB) in an advanced NSAH model of FGF21KO mice with HFMCD feeding for 3 months. Unlike the early stage NASH model, the gross appearance of in advanced NASH liver showed diffusely pale-yellow color lobes and increased liver weight along with increased glucose tolerance, liver weight, body weight, and serum TG level in FGF21KO-HFMCD mice (Figure S2). Of note, when we analyzed the morphological changes of NASH liver, multiple nodules were detected microscopically in hepatic parenchyma of the liver tissues from FGF21KO+HFMCD mice (Figure 4A). Histological changes showed severe steatohepatitis, as evident by significantly increased NAS, multiple nodules in hepatic parenchyma, significantly increased for Kupffer cells/macrophages detected by IHC for F4/80, and significantly increased serum ALT level in the FGF21KO-HFMCD mice compared to all other groups (Figure 4A). As expected, significantly increased protein levels of FGF15 and FGFR4/β-klotho and were detected by Western blot analysis in the liver of FGF21KO-HFMCD mice compared to all other groups (Figure 4B). Overexpressions of FGFR4 and β-klotho in the liver of FGF21KO-HFMCD mice with advanced NASH were confirmed by IHC and qPCR (Figure S3). Taken together, continuously up-regulated FGFR4 expression in advanced NASH should call attention because hyperactivation of FGFR4 by FGF19 was reported in colon cancer and HCC 37. However, FGF15/19 was also reported to down-regulate FGFR4 and β-klotho 27. Therefore, we further studied the FGFR4 signaling in advanced NASH model in regard of the malignant potential.

### Up-regulated FGFR4 signaling is coupled to fibrotic and malignant events in FGF21KO mice

**Figure 5:**
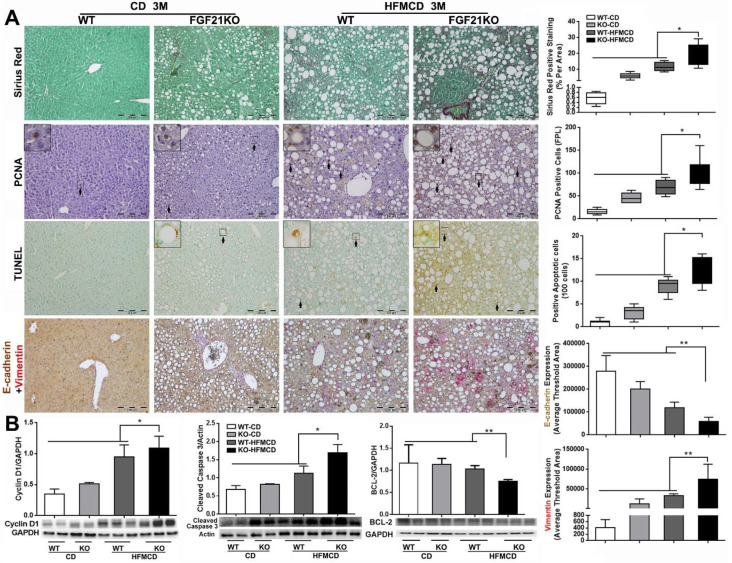
** A:** Representative images of Sirius Red staining, PCNA and TUNEL by IHC staining, E-cadherin/Vimentin by a dual IHC staining along with computer-imaging analysis in liver tissues from advanced NASH model and controls. **B:** Western blot analysis for the protein levels of cyclin D1, cleaved caspase-3 and BCL-2 in liver tissues from advanced NASH model and controls. WT: wild type; KO: FGF21KO; CD: control diet; and HFMCD: high fat methionine-choline deficient. FPL: field per low power. *, p<0.05; **, p<0.01.

Cirrhosis and HCC have become the major liver-related clinical endpoints in NASH, while fibrosis progression and malignant transformation are driven by repetitive damages/repairs via apoptosis and cell proliferation 2. To study fibrosis and the cellular events, we performed Sirius Red staining for fibrosis, IHC of PCNA for proliferation, and TUNEL assay for apoptosis in the liver tissues from the advanced NASH model with HFMCD feeding for 3 months. Significantly increased level of collagen fiber, as showing the red color by Sirius Red staining, was found in FGF21KO-HFMCD mice compared to all other groups (Figure 5A). Consistent to Sirius Red staining, significantly increased levels of apoptosis and cell proliferation, indicated by the indexes of positive apoptotic cells and positive PCNA cells, were also found in FGF21KO-HFMCD mice compared to all other groups (Figure 5A). To evaluate the potential malignant phenotype, the epithelial-mesenchymal transition (EMT) event was investigated by a dual IHC staining for E-cadherin and vimentin in the liver tissues. The results indicated that E-cadherin expression was significantly down-regulated but vimentin expression was significantly up-regulated in the FGF21KO-HFMCD mice, compared to all other groups (Figure 5A). Western blot analysis was further performed in the liver tissues and the results indicated that significant increases of cyclin D1 and cleaved caspase-3 but significant decrease of BCL-2 was found in FGF21KO-HFMCD mice, compared to all other groups (Figure 5B). Taken together, increased fibrosis and deleterious molecular and cellular events were found in in FGF21KO-HFMCD mice which with advanced NASH, while up-regulated FGFR4 expression was coupled to these molecular and cellular events.

### Blockage of FGFR4 attenuates proliferation and EMT in hepatocytes and HCC cells

**Figure 6:**
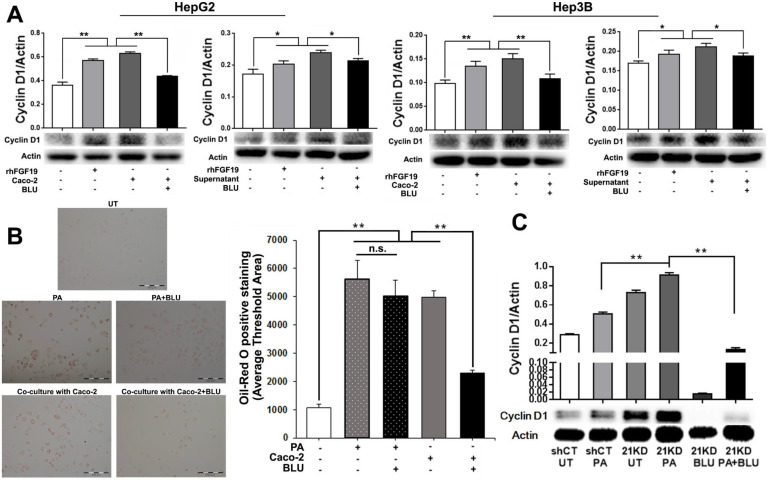
** A:** Western blot analysis for cyclin D1 protein levels in HepG2 cells and Hep3B cells co-cultured with Caco-2 cells, with rhFGF19 treatment at 100ng/ml for 24 hours, with co-culture supernatant treatment for 24 hours, and with BLU9931 treatment at 100nM for 24 hours. **B:** Lipid accumulation detected by Oil Red O staining along with computer-imaging analysis in Hep3B cells challenged with PA (palmic acid) at 100uM for 48 hours, and treated with BLU9931 treatment at 100nM for 24 hours. **C:** Western blot analysis for the protein levels of cyclin D1 in shCT-FL83B cells and FGF21KD-FL83B cells challenged with PA at 100uM for 24 hours, and treated with BLU9931 at 100nM for 24 hours. UT: untreated; PA: palmic acid; shCT: shCT-FL83B cells; 21KD: FGF21KD-FL83B cells. n.s., no significance; *, p<0.05; **, p<0.01.

**Figure 7:**
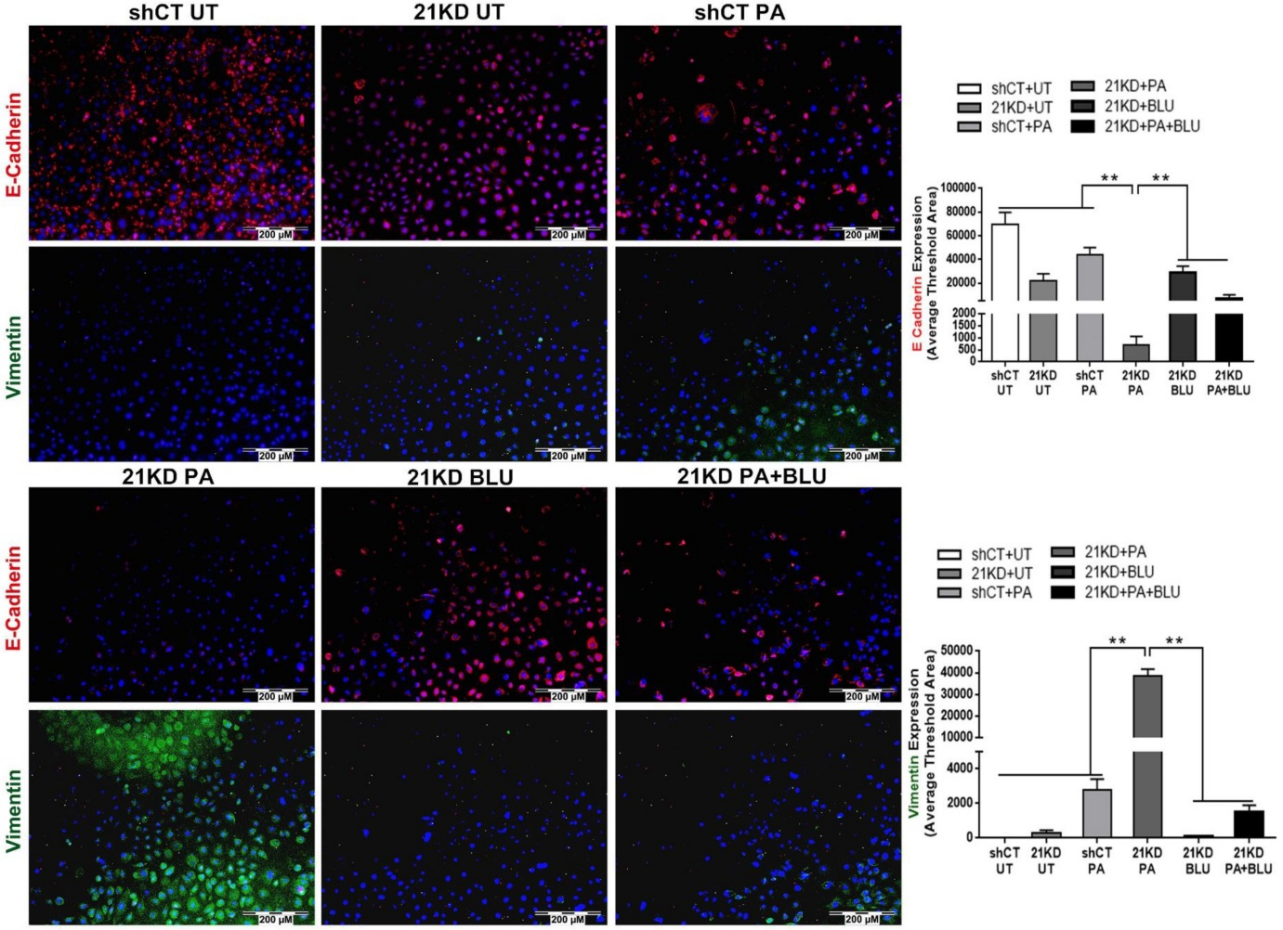
E-cadherin/Vimentin expressions by immunofluorescent staining in shCT-FL83B cells and FGF21KD-FL83B cells challenged with PA at 100uM for 24 hours, and treated with BLU9931 at 100nM for 24 hours. UT: untreated; PA: palmic acid; shCT: shCT-FL83B cells; 21KD: FGF21KD-FL83B cells. *, p<0.05; **, p<0.01.

To mimic the enterohepatic circulation in regards of the FGF19/FGFR4 signaling, we further performed a co-cultured study using human hepatocyte cell lines (HepG2 and Hep3B) and an enterocyte cell line (Caco-2). When Caco-2 cells co-cultured with either HepG2 or Hep3B, significantly up-regulated FGF19 mRNA was detected in the Caco-2 cells (Figure S4). BLU9931, a highly selective, covalent, small-molecule inhibitor that specifically targets FGFR4, binds within the ATP-binding pocket of FGFR4, forming a covalent bond with Cys552 to specific targeting to FGFR4 29. By suing BLU9931, we further performed the studies to investigate whether inhibiting FGFR4 could abolish the FGF19/FGFR4 signaling thereby alleviate the molecular and cellular events related to the NASH pathogenesis. Consistent with the results of co-culture supernatant treatment and rhFGF19 treatment, co-culture with Caco-2 cells significantly increased levels of cyclin D1, while BLU9931 treatment attenuated the up-regulated cyclin D1 levels in both HepG2 cells and Hep3B cells (Figure 6A). The result of Oil Red O staining indicated significantly increased levels of lipid accumulation in Hep3B cells either co-cultured with Caco-2 cells or PA treatment, while BLU9931 treatment attenuated the increased levels of lipid accumulation with statistical significance (Figure 6B). With BLU9931 treatment, significantly decreased protein level of cyclin D1 was also found in FGF21KD FL83B cells either with PA challenging or without (Figure 6C). As EMT has been widely accepted as a cellular event to be associated with tumor initiation, we further used BLU9931 to study whether blocking FGFR4 could inhibit EMT event in FGF21KD FL83B cells either with PA challenging or without. Consistently, blockage of FGFR4 signaling alleviated significantly the EMT in FGF21KD FL83B cells either with PA challenging or without (Figure 7). These results demonstrated that blockage of FGFR4 could attenuate the deleterious cellular and molecular events which might be associated with NASH development and NASH-HCC progression.

## Discussion

As a liver safeguard 38, FGF21 was widely reported to alleviate hepatic fat stress via directly reducing hepatic lipid accumulation in an insulin-independent manner 39. Similar to FGF21, FGF15/19 was also reported to function in controlling whole body lipid metabolism through increasing energy expenditure, FA oxidation, and decreasing de-novo lipogenesis 20. However, it was unknown whether FGF15/19 could directly reduce hepatic lipid accumulation, especially when FGF21 was not function well, i.e., FGF21 resistance in obesity 40. In this study, significant increase of FGF15 production was found in the NASH-FGF21KO mice. As previously reported 10, both of FGF15/19 and FGF21 involved in the biological process of lipid metabolism but the modulating pattern showed different. FGF21 can be rapidly produced in hepatocytes by fasting in mice while FGF15/19 is secreted from enterocytes upon bile acids stimulation. The possible reasons for overexpression of FGF15 in NASH-FGF21KO mice could be that: 1) the high fat diet caused over-secreted bile acids; 2) the severity of NASH in FGF21KO mice caused overexpression of FGF15 to compensate the FGF21. Although overexpression of FGF15 was found NASH-FGF21KO mice, the *in vitro* study showed that rmFGF15 treatment was unable to alleviate the lipid accumulation in FGF21KO-FL83B cells as evidenced by Oil Red O staining. In contrast, the up-regulated expressions of FGF15 and FGFR4 were coupled to fibrosis, hepatocyte injury/repair, and potential malignant events in the FGF21KO mice with advanced NASH.

Both FGF15 and FGF19 function as a negative feedback loop shutting down BA synthesis when BAs levels are high in the intestinal mucosa. Regarding the bioactivity on NASH, studies either from transgenic mice or treatment with FGF19 protein have shown that FGF19 alleviates lipid accumulation in the liver and thereby prevents NASH 20, 41, 42. However, the effect of FGF15 on NASH is reportedly contradictory. For example, a study reported that the FGF15 knockout mice fed a HFD worsened steatosis 20 but another study that also fed with HFD to the FGF15 knockout mice did not show worsened steatosis severity^43^. The protein discrepancy between FGF15 and FGF19 has been identified previously, in which they share only 50% sequence homology 30, 44 even though they are orthologs and both are considered as endocrine FGFs because they do not bind heparin sulfate and thus can escape extracellular matrix. Studies using chimeric immunodepressed mice transplanted with human hepatocytes further emphasized the bioactive discrepancy between FGF15 and FGF19, in which FGF19 administration reversed the enlarged bile acid pool size 45, but significantly elevated FGF15 was unable to suppress hepatic CYP7A1 expression 33. The increased hepatic BAs level not only induces liver injury 46 but also plays an important role in the regulation of hepatocytes regeneration 47, contributing to NASH development through repetitive injury/repair. BAs reabsorbed in the intestine can increase the FGFR4/β-klotho levels in hepatocytes for subsequent FGF15/FGFR4 signaling in liver 48, while hepatic FGFR4 is reported to promote hepatic lipid accumulation by either HFD or healthy diet 24, 36. In our study, the significant increased FGF15 in the NASH-FGF21KO mice was unable to alleviate hepatic lipid accumulation, while up-regulated FGFR4 expression was coupled to fibrosis and deleterious molecular and cellular events in advanced NASH. This might be explained by, 1) although FGF15 protein was very high in liver, it was unable to suppress hepatic CYP7A1 expression 33 for BA synthesis and BAs could induce liver injury and cell death 46; 2) the BAs mediated up-regulation of FGFR4 in hepatocytes 48 could not only promote hepatic lipid accumulation 24, 36 but also induce regeneration 47, causing repetitive injury/repair in liver; and 3) lack of FGF21 further worsened steatosis and the NASH progression. Our findings indicate a dramatically different roles of FGF15 and FGFR4-β-klotho during NASH development in FGF21KO mice.

The liver generally maintains an appropriate size in adults. Loss of hepatocytes because of repetitive injury allows the liver to begin growing which is accepted as a fundamental mechanism(s) in cancer biology. Emerging studies have shown that FGFR4 play important roles in liver regeneration and carcinogenesis. For post-hepatectomy liver regeneration, FGFR4 was found to complex with β-klotho on hepatocyte membrane, upon FGF19/FGF15 binding, to initiate regeneration and orchestrate BAs acid detoxification as a protective mechanism in concurrence with the proliferative signaling 49. In HCC, FGF19 elicits cell proliferation 50 and promotes the HCC cell survival and increased resistance to apoptosis via activating a FGFR4-GSK3β-Nrf2 signaling cascade 51. The aberrant FGFR4 signaling has been reported to be an oncogenic-driver pathway for HCC development 52. In our studies, up-regulated FGF15 and FGFR4/β-klotho were found to coupled fibrosis and deleterious cellular events during the NASH development in FGF21KO mice. These findings are in accordance with our previous studies in HFD- or diabetes- induced NASH mice 6, 8. This study is mainly designed to investigate FGF15/FGFR4 expression during the NASH development in FGF21KO mice. The major limitation of this study is lack of FGF15KO/overexpressing mice to elucidate the molecular mechanisms of FGF15/FGFR4 signaling pathway contributing to NASH progression and HCC development. Further studies are needed to determine the roles of FGF15/FGFR4/β-klotho as well as the downstream signaling during NASH progression. The FGFR4/β-klotho/BAs associated carcinogenesis via Gut-Liver axis is also an important issues and needs to further study in NASH-HCC transition models.

## Conclusion

The increased FGF15 production in NASH-FGF21KO mice could not substitute for FGF21 to compensate its lipid metabolic benefits thereby to prevent NASH development. The up-regulated FGFR4 signaling was coupled to cellular and molecular events which might associate to carcinogenic transformation. This study provided a new insight into FGF15 and FGFR4 signaling during NASH development and the potential pharmacological application to target FGFR4 for treatment in advanced NASH.

## Supplementary Material

Supplementary figures and tables.

## Abbreviations

FGF21: Fibroblast growth factor21
FGF15/19: Fibroblast growth factor15/19
NASH: Nonalcoholic steatohepatitis
HFMCD: High fat methionine-choline deficient
FFA: free fatty acid
FA: fatty acid
KO: knockout
NAFLD: Non-alcoholic fatty liver disease
HCC: Hepatocellular carcinoma
ALT: Alanine aminotransferase
TG: Triglyceride
H&E: Hematoxylin and eosin
NAS: NAFLD activity score
shCT: shRNA Control
PA: Palmic acid
