# Supplementary material for: Aberrant FGFR4 signaling worsens nonalcoholic steatohepatitis in FGF21KO mice
Source: Int J Biol Sci. 2021 Jun 22;17(10):2576–89. doi: 10.7150/ijbs.58776 (PMC8315028; doi:10.7150/ijbs.58776)
Supplement: Supplementary file 1 — Supplementary figures and tables. [file ijbsv17p2576s1.pdf]

**Supplementary materials**

| Table 1 | *List of antibodies* |
|---|---|
| Antibody | Source (catalog) |
| FGF15 | Santa Cruz (sc-398338) |
| FGF21 | Abcam (ab64857) |
| KLB | Abcam (ab233416) |
| FGFR4 | Abcam (ab178396, ab44971) |
| Cleaved caspase-3 | Cell Signaling Technology (cst9664t) |
| Bcl-2 | Cell Signaling Technology (cst3498s) |
| Cyclin D1 | Cell Signaling Technology (cst2978) |
| E Cadherin | Abcam (ab76055) |
| Vimentin | Abcam (ab92547) |
| PCNA | Abcam (ab15497) |
| F4/80 | BD Biosciences (565409) |
| GAPDH | Cell Signaling Technology (cst5174s) |
| β-Actin | Cell Signaling Technology (cst3700s) |
| Anti-mouse IgG | Cell Signaling Technology (cst7076S) |
| Anti-rabbit IgG | Cell Signaling Technology (cst7074S) |
| Anti-rat IgG-HRP | Abcam (ab97057) |
| Alexa Fluor™ 488 | Invitrogen™ (A11053, A11054) |
| Alexa Fluor® 568 | Invitrogen™(A11066) |

| Table 2 | | Primers of the target genes | |
|---|---|---|---|
| Name | Gene ID | Sequence (5‘-3’) mouse & human | |
| KLB | 83379 | Forward | 5'- AGGCTCTGAAAGCATATCTC-3' |
| | | Reverse | 5'- GCTTAGATTTCTCTTCAGTCAG -3' |
| FGFR4 | 14186 | Forward | 5'-GCCTCCGACAAGGATTTGGCA -3' |
| | | Reverse | 5'-GAGTGCAGACACCCAGCAGGT -3' |
| FGF15 | 14170 | Forward | 5'-GAGGACCAAAACGAACGAAATT -3' |
| | | Reverse | 5'- ACGTCCTTGATGGCAATCG-3' |
| FGF21 | 56636 | Forward | 5'-ATGGAATGGATGAGATCTAGAGTTGG -3' |
| | | Reverse | 5'-TCTTGGTGGTCATCTGTGTAGAGG -3' |
| GAPDH | 14433 | Forward | 5'-GTGAAGGTCGGTGTGAACGGATT-3' |
| | | Reverse | 5'-CGTGAGTGGAGTCATACTGGAACAT-3' |
| FGF19 (h) | 9965 | Forward | 5'-CAGCTGTACAAGAACAGAGGCTTTC -3' |
| | | Reverse | 5'-AAATGGGTCCATGCTGTCGGTCTCC -3' |
| GAPDH (h) | 2597 | Forward | 5'-TCCCATCACCATCTTCCAG -3' |
| | | Reverse | 5'-GAGTCCTTCCACGATACCAA -3' |

**Supplementary Methods**

Establishment of FGF21 knockdown cell lines

A mouse liver cell line, FL83B (ATCC CRL-2390), and a mouse hepatoma cell line, Hepa1-6 (ATCC CRL-1830), were used for the in vitro study. To establish FGF21 gene knock down (FGF21KD) cell lines, fourth generation lentivirus packing system (Lenti-X, Takara-Clontech) was used to generate FGF21 gene knock down (FGF21KD) cell lines of Hepa1-6 and FL83B. Briefly, puromycin-resistant shFGF21 (Sigma-Aldrich, MO) vectors were obtained, amplified, and purified for packing. For generating lentivirus, the lenti-X reagent was mixed with 6 μg of shRNA plasmid and transfected in 293T cell line for packing. The harvested lentivirus was used to transduced target the two cell lines using polybrene with established protocol. The cells were transduced with shRNA targeting FGF21 (shFGF21, **Sequence:** CCGGCTCTACACAGATGACGACCAACTCGAGTTGGTCGTCATCTGTGTAGAGTTTTTG) (NM_020013/TRCN0000067373, Sigma-Aldrich), or sham sequences (shControl) (SHC002, Sigma-Aldrich) according to manufacturer's instructions. The Hepa1-6-21KD and the Hepa1-6-shControl (shCT) cells were cultured in DMEM (Gibco, USA), while the FL83B-21KD and FL83B-shCT cells were cultured in F12K medium (ATCC® 30-2004™). The FGF21KD cell lines were selected by puromycin (Sigma-Aldrich) and expanded after confirmation of gene knockdown by qRT-PCR and Western blot analysis.

**Supplementary Figures**

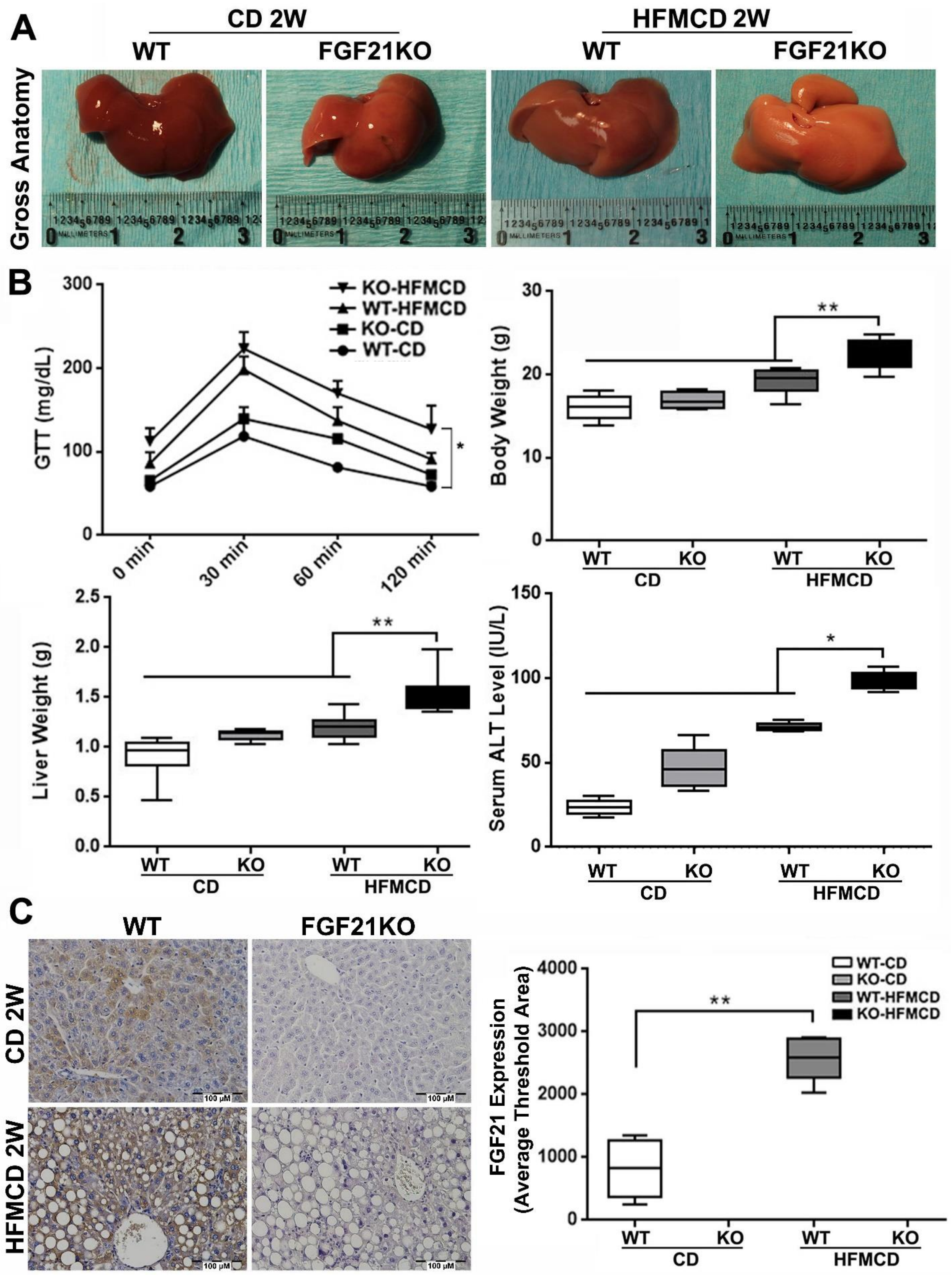


**Figure S1**. **A:** Representative gross anatomy of liver lobes from the early NASH model and

controls. **B:** glucose tolerance test, body weight, liver weight, and serum ALT levels from the early NASH model and controls. **C:** FGF21 expression by IHC staining along with computer-imaging analysis in the liver tissues from early NASH model and controls. Scale bar=100 µm. GTT: glucose tolerance test; WT: wild type; KO: FGF21KO; CD: control diet; HFMCD: high fat methionine-choline deficient. *, P<0.05; **, P<0.01.

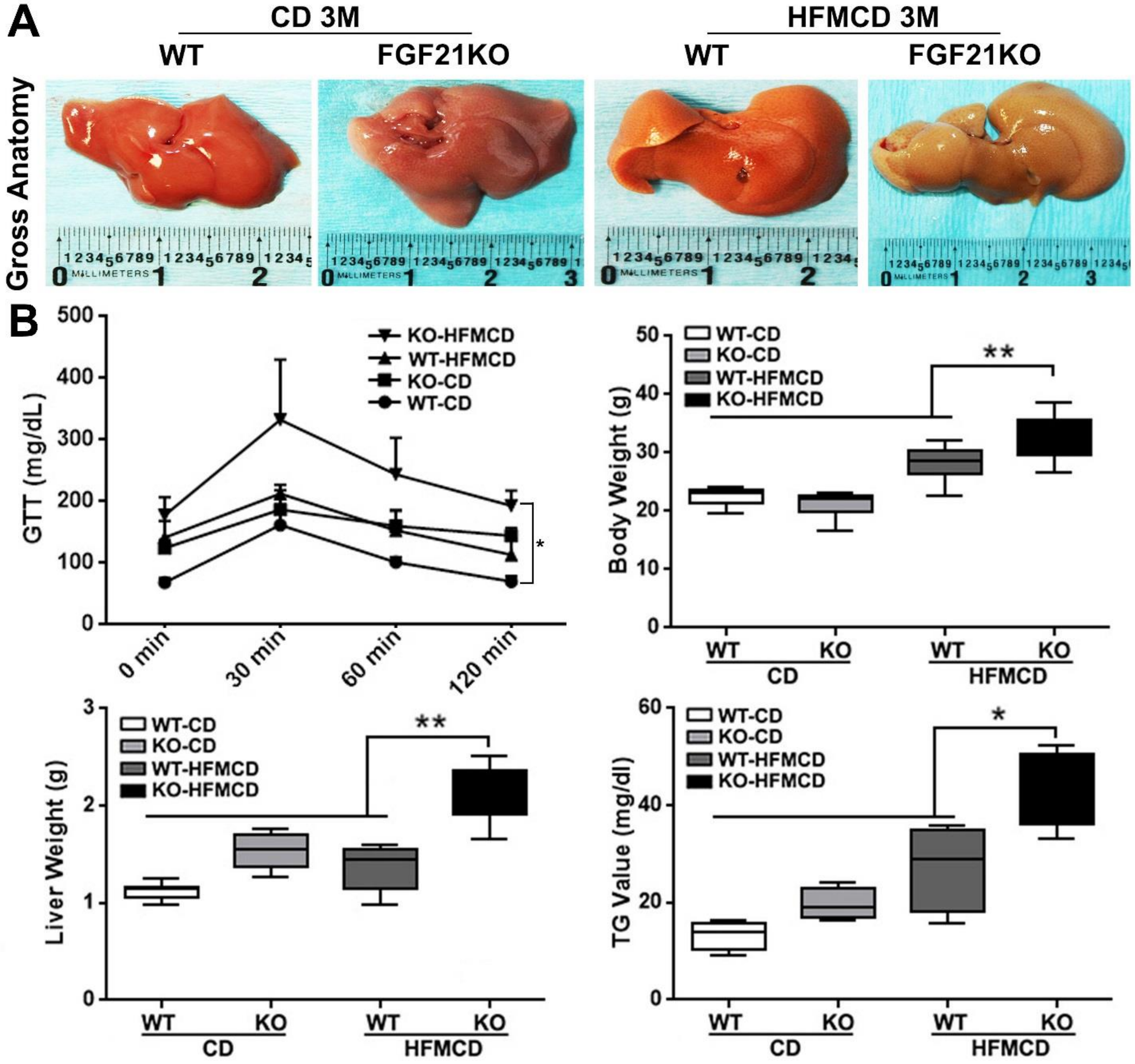


**Figure S2**. **A:** Representative gross anatomy of liver lobes from the advanced NASH model and controls. **B:** glucose tolerance test, Body weight, liver weight, and serum TG levels from the advanced NASH model and controls. GTT: glucose tolerance test: GTT; WT: wild type; KO: FGF21KO; CD: control diet; HFMCD: high fat methionine-choline deficient. *, P<0.05; **, P<0.01.

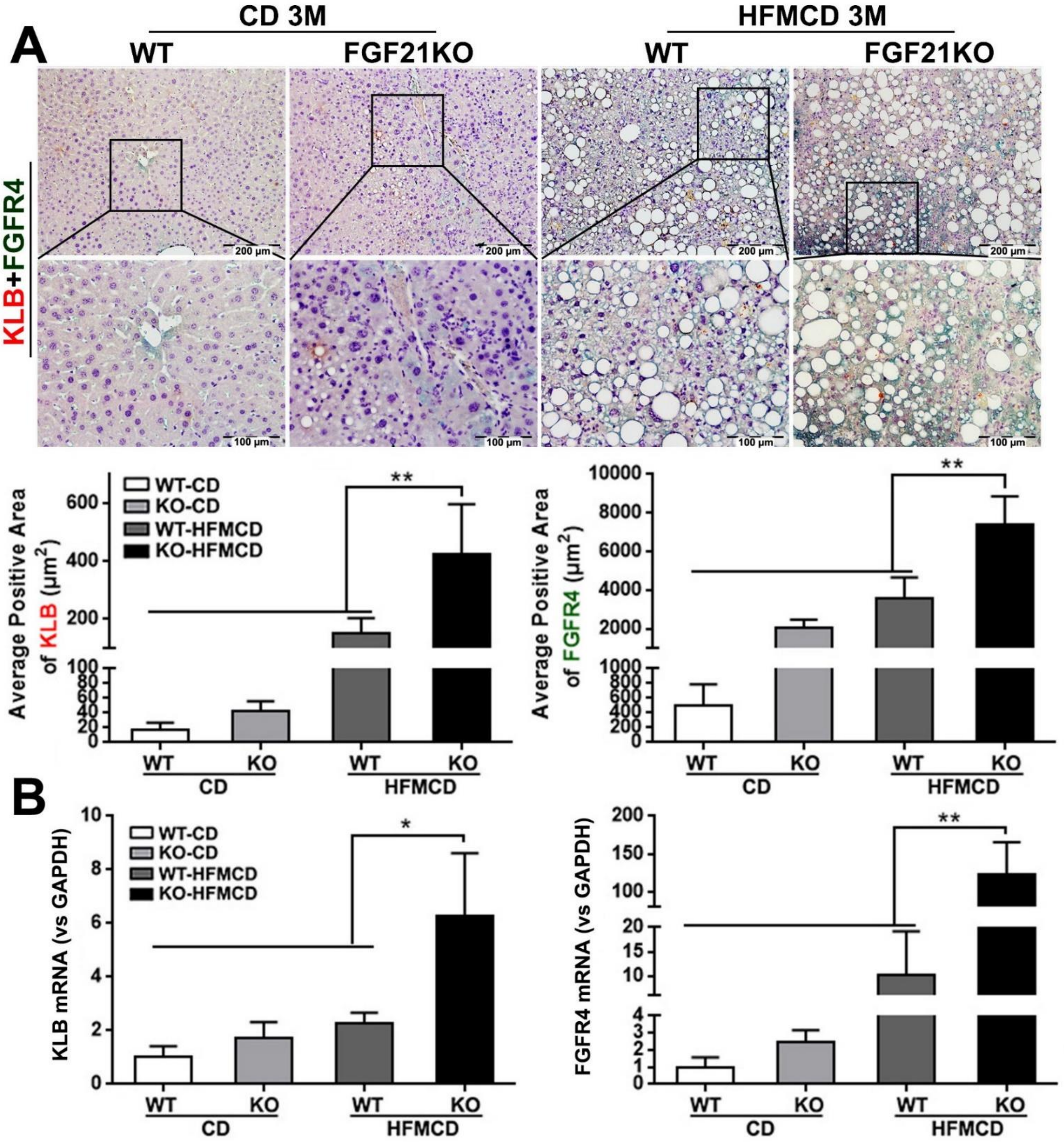


**Figure S3**. **A:** Representative images of FGFR4/β-klotho expression by a dual IHC staining along with computer-imaging analysis in liver tissues from advanced NASH model and controls. The dual IHC staining showed that FGFR4 and β-klotho were co-expressed and significantly up-regulated in the hepatocytes of FGF21KO-HFMCD mice, compared to all other groups. Scale bar=200 μm. **B:** mRNA expressions of FGFR4/β-klotho by qPCR. WT: wild type; KO: FGF21KO; CD: control diet; HFMCD: high fat methionine-choline deficient. *, $P<0.05$; **, $P<0.01$.

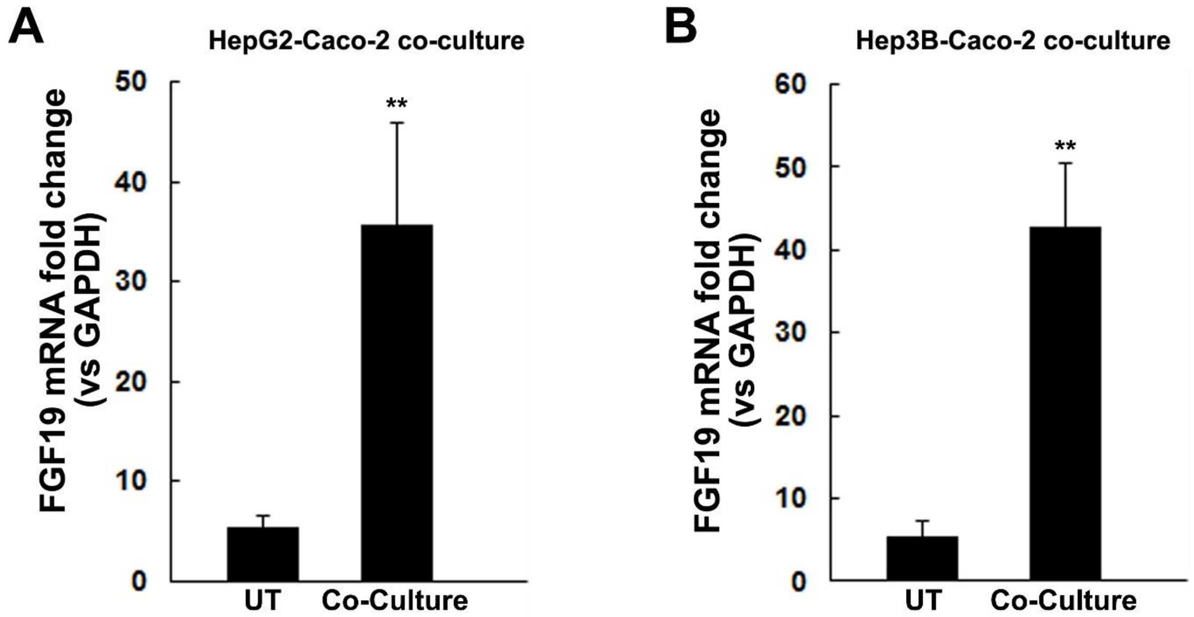


**Figure S4**. mRNA expressions of FGF19 by qPCR. WT: wild type; UT: untreated. **, P<0.01 vs UT.
